# An Integrated Study Based on UPLC-QTOF/MS Network Pharmacology and In Vivo Validation of the Anti-Obesity Effects of the 60% Ethanol-Eluted Fraction from *Rheum tanguticum*

**DOI:** 10.3390/plants15121858

**Published:** 2026-06-16

**Authors:** Ming Wang, Xiaoli Wu, Yajun Li, Xinruo Wei, Chuan Luo, Chen Chen

**Affiliations:** 1 Anhui Provincial Engineering Laboratory for Efficient Utilization of Featured Resource Plants, College of Life Sciences, Huaibei Normal University, Huaibei 235000, China; 18895631256@163.com (M.W.); 18298065221@163.com (X.W.); 17856116589@163.com (X.W.); 2 Anhui Key Laboratory of Traditional Chinese Medicine Formula Granules, Anhui Huaren Jinchan Pharmaceutical Co., Ltd., Huaibei 235100, China; liyajun@999.com.cn; 3 Chinese Academy of Sciences Key Laboratory of Tibetan Medicine Research, Northwest Institute of Plateau Biology, Xining 810008, China

**Keywords:** *Rheum tanguticum* Maxim. ex Balf., obesity, UPLC-QTOF/MS, network pharmacology, lipid metabolism

## Abstract

Obesity has emerged as a significant global public health challenge, yet the clinical utility of existing anti-obesity drugs is often constrained by limited efficacy and adverse safety profiles. *Rheum tanguticum* Maxim. ex Balf., a traditional medicinal plant, has shown potential in modulating glucose and lipid metabolism; however, its specific anti-obesity mechanisms remain poorly characterized. In this study, the chemical profile of the 60% ethanol-eluted fraction of *R. tanguticum* (RTE) was characterized via UPLC-QTOF/MS, followed by network pharmacology analysis to predict regulatory targets and enriched pathways. Subsequently, a high-fat diet (HFD)-induced obese mouse model was established to evaluate the anti-obesity effects of RTE by monitoring body weight, Lee’s index, fat-to-body weight ratio, serum lipid profiles, and liver histopathological changes. A total of 14 major compounds, primarily anthraquinone glycosides, were identified. Integrated network analysis identified 10 hub targets, including TNF, EGFR, and TP53. In vivo experiments demonstrated that RTE significantly attenuated body weight gain and reduced Lee’s index, fat-to-body ratios, and serum levels of TC, TG, and LDL-C. Furthermore, RTE treatment markedly alleviated hepatic steatosis and inflammatory infiltration in obese mice. These findings suggest that RTE exerts potent anti-obesity effects through a multi-target and multi-pathway mechanism that regulates lipid metabolism and suppresses inflammation. This study improves our understanding of the pharmacological value of *R. tanguticum* and provides a scientific basis for its development as a functional food ingredient or therapeutic agent against obesity.

## 1. Introduction

Obesity is a chronic metabolic disease arising from an imbalance in energy homeostasis and a major contributor to type 2 diabetes, nonalcoholic fatty liver disease, and cardiovascular and cerebrovascular disorders [1]. In recent years, shifts in lifestyle and dietary patterns have driven a sustained rise in the global prevalence of obesity. Current estimates indicate that over two billion individuals worldwide are overweight or obese, with a further substantial increase projected by 2030 [2,3,4]. Although several anti-obesity drugs, such as orlistat, liraglutide, and semaglutide, have been introduced into clinical practice and achieve pronounced short-term weight loss, they are frequently accompanied by non-negligible adverse effects including gastrointestinal intolerance, cardiovascular risk, and central nervous system toxicity. Bariatric surgery, although efficacious, is limited by its invasiveness, high cost, and postoperative complications, restricting its broader clinical application. Against this backdrop, natural products have attracted increasing scientific attention owing to their favorable safety profiles and multi-target regulatory potential. The development of novel natural anti-obesity agents that integrate safety, efficacy, and long-term intervention potential has thus become a key research priority.

Traditional Chinese medicine has a long history of utilizing botanical formulations to manage metabolic disorders. Modern pharmacological studies indicate that these herbal treatments often exert systemic anti-obesity effects through a “multi-component and multi-target” synergistic approach, offering unique advantages in regulating complex metabolic networks [5,6]. *Rheum tanguticum* Maxim. ex Balf. (Polygonaceae) is one of the three official rhubarb species documented in the Chinese Pharmacopoeia; its dried root and rhizome serve as a frequently used medicinal part for obesity management [7,8,9,10,11]. Contemporary pharmacological investigations reveal that its active constituents not only provide laxative actions but also promote steroid excretion, suppress cholesterol accumulation, and exhibit potent anti-inflammatory, lipid-lowering, and hypoglycemic activities [12,13,14,15,16,17].

Building on these foundational findings, modern pharmacological studies have increasingly focused on the specific molecular mechanisms of *R. tanguticum*, identifying anthraquinones—such as emodin, rhein, and chrysophanol—as its principal bioactive constituents. Recent evidence suggests that these anthraquinone derivatives possess profound metabolic-regulatory properties. For instance, emodin has been reported to ameliorate dyslipidemia and hepatic steatosis by activating the AMPK signaling pathway, while rhein exhibits pronounced anti-inflammatory effects by inhibiting macrophage infiltration in adipose tissue [18,19,20].

While the metabolic benefits of individual anthraquinones are clear, how the complete *R. tanguticum* extract functions in vivo remain poorly understood. Obesity is driven by multiple overlapping pathways, making it necessary to evaluate this botanical extract as a complete, multi-component system rather than focusing on single molecules. In this study, we first profiled the chemical constituents of the *R. tanguticum* extract via UPLC-QTOF/MS. We then combined network pharmacology predictions with an in vivo high-fat diet (HFD) mouse model to map out its regulatory network. Ultimately, this work clarifies the synergistic mechanisms driving the extract’s anti-obesity effects, offering direct biological evidence for its application in metabolic interventions.

## 2. Results

### 2.1. UPLC-QTOF-MS Analysis

**Figure 1:**
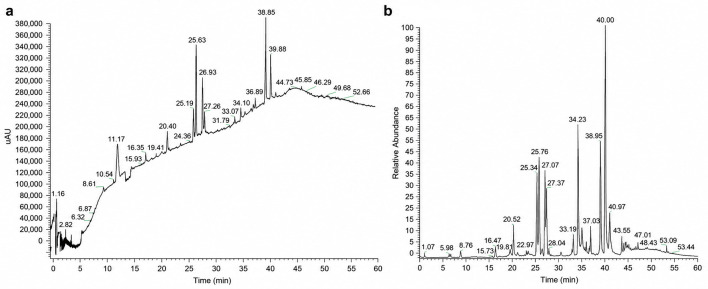
Chromatogram of components from ethanol extract of *Rheum tanguticum*. (**a**) Chromatogram at 280 nm; (**b**) Mass spectrum.

The 60% ethanol-eluted fraction of *R. tanguticum* (RTE) was analyzed by UPLC-QTOF-MS, and the representative chromatogram and total ion chromatogram are shown in Figure 1. Figure 1a presents the chromatogram, whereas Figure 1b shows the total ion chromatogram. Compounds were tentatively identified by extracting MS data and fragmentation patterns from the total ion chromatogram and matching retention times with MS information for secondary metabolites. In total, 14 compounds were identified (Table 1), including 10 anthraquinone derivatives and their glycosides; namely, emodin-1-O-glucoside, emodin-3-O-glucoside, emodin-6-O-glucoside, emodin-8-O-glucoside, chrysophanol-1-O-glucoside, chrysophanol-8-O-glucoside, physcion-8-O-glucoside, physcion-8-O-diglucoside, physcion-1-O-diglucoside, and rhein. Two phenylbutanoid compounds and one phenolic acid derivative were also identified.

### 2.2. Network Pharmacology

#### 2.2.1. Selection of Putative Targets for RTE and Obesity

**Figure 2:**
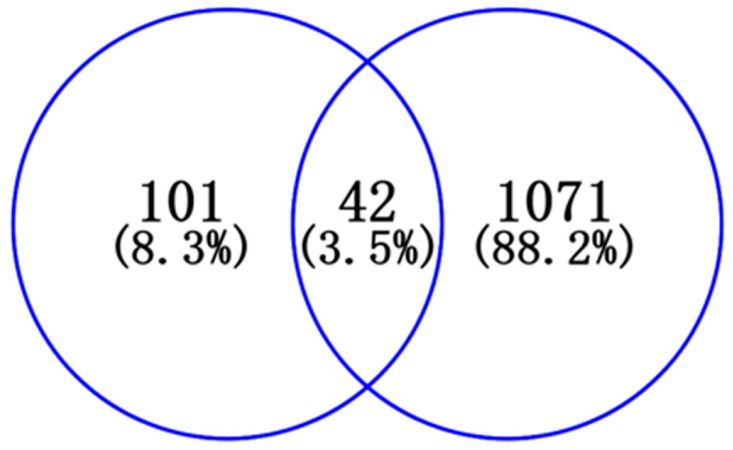
The Venn diagram of genes and disease.

Based on the compounds identified by UPLC-QTOF-MS, 101 targets associated with the RTE constituents were retrieved from PubChem and SwissTargetPrediction. In parallel, obesity-related targets were collected from GeneCards, NCBI Gene, and DisGeNET using “obesity” as the keyword, yielding 1071 disease-related genes after removing duplicates. Intersecting the compound-related and disease-related targets in Venny 2.1 identified 42 shared targets (Figure 2).

#### 2.2.2. Prediction of Key Targets

**Figure 3:**
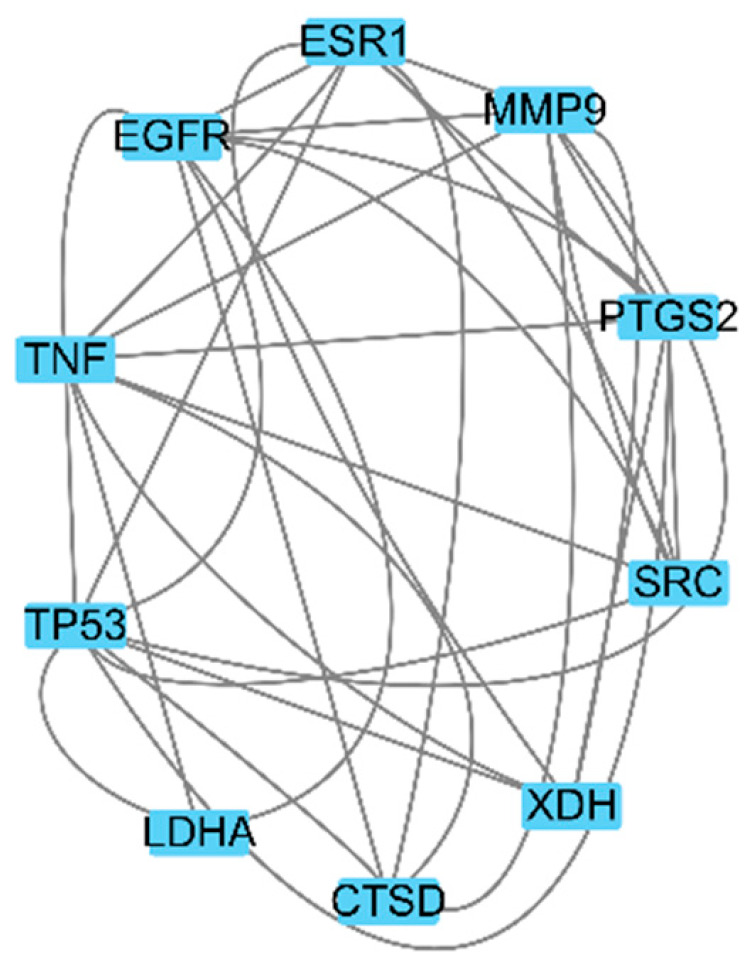
Obesity PPI network diagram.

The shared targets were imported into STRING for protein–protein interaction (PPI) network analysis. Topological analysis was performed using NetworkAnalyzer, and genes with degree values above the mean were selected as key targets. TNF, EGFR, TP53, LDHA, CTSD, XDH, SRC, PTGS2, MMP9, and ESR1 were identified as the 10 most prominent key targets. The resulting core PPI network contained 10 nodes and 34 edges (Figure 3).

#### 2.2.3. GO and KEGG Enrichment Analysis

**Figure 4:**
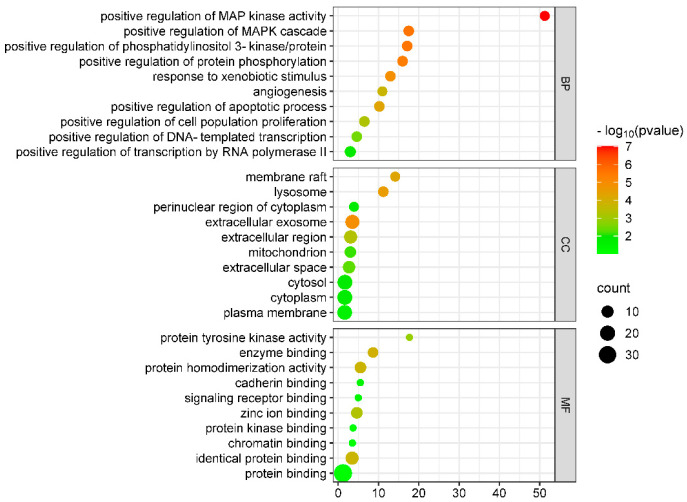
GO enrichment analysis plot.

GO and KEGG enrichment analyses were conducted to further elucidate the biological functions of the core targets. GO analysis yielded 212 enriched biological process terms, 44 molecular function terms, and 39 cellular component terms (Figure 4). In the biological process category, the targets were significantly enriched (*p* < 0.05) in positive regulation of cell signaling cascades, particularly the MAPK cascade, MAP kinase activity, and positive regulation of phosphatidylinositol 3-kinase signaling, as well as responses to exogenous stimuli. In the cellular component category, the targets were mainly associated with extracellular vesicles, membrane rafts, the plasma membrane, cytoplasm, and cytosol. In the molecular function category, the targets were mainly involved in protein binding, homodimeric protein binding, protein homodimerization activity, zinc ion binding, and enzyme binding.

**Figure 5:**
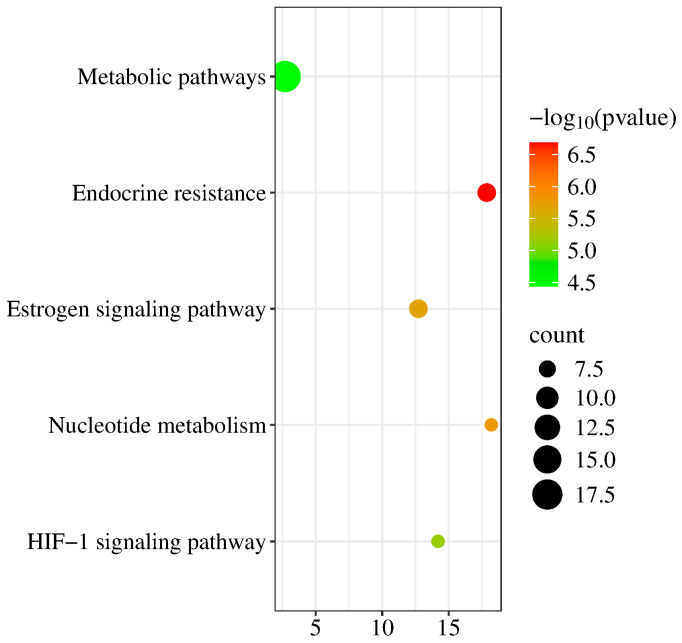
Bubble plot of the top 5 enriched KEGG pathways.

To further elucidate the underlying biological mechanisms of RTE against obesity, KEGG pathway enrichment analysis was performed on the core intersecting targets. As shown in Figure 5, the analysis revealed that the core targets were highly enriched in several critical metabolic and inflammatory networks. The top 5 significantly enriched pathways were Metabolic pathways, Endocrine resistance, Estrogen signaling pathway, Nucleotide metabolism, and the HIF-1 signaling pathway.

#### 2.2.4. Network Analysis of the Component–Target–Obesity–Pathway Network

**Figure 6:**
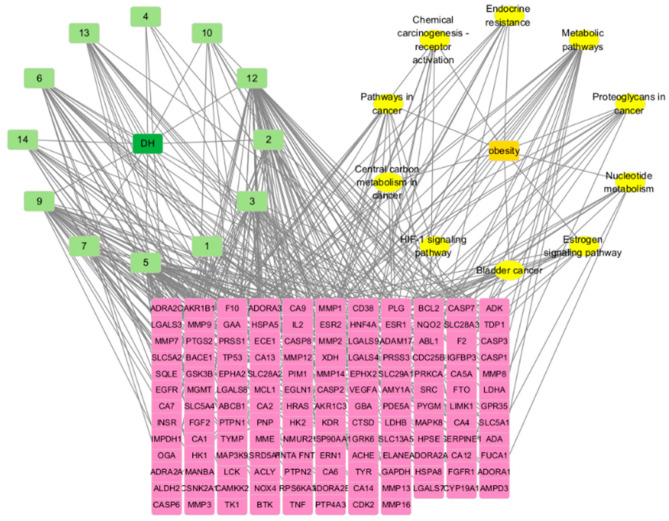
Component–disease–pathway–target network diagram of the 60% ethanol-eluted fraction of *R. tanguticum* (RTE). Green nodes represent active compounds, red nodes represent potential targets, and yellow nodes represent enriched signaling pathways. The edges indicate the interactions among compounds, targets, and pathways.

To further elucidate the potential molecular mechanism underlying the anti-obesity effect of RTE, the previously constructed component–target–obesity–pathway network was imported into Cytoscape 3.8.0 for visualization (Figure 6). The network clearly illustrates a synergistic regulatory mode involving multiple components, multiple targets, and multiple pathways in the modulation of obesity.

### 2.3. Acute Toxicity and Anti-Obesity Effects of RTE

#### 2.3.1. Acute Toxicity Evaluation of RTE

In the acute toxicity test, no deaths or overt signs of clinical toxicity were observed in mice within the 14-day observation period after oral gavage of the RTE fraction. These results indicate that the oral LD50 of this fraction was >5000 mg/kg. According to standard toxicity classification criteria, the fraction can be considered practically non-toxic or of low toxicity, indicating good safety at the doses used in this study.

#### 2.3.2. Effects on Body Weight

As shown in Table 2, body weight changes differed markedly among the groups. Compared with the normal control group, body weight gain was significantly higher in the model control group (*p* < 0.05). In contrast, body weight gain was significantly lower in the high- and middle-dose RTE groups than in the model control group (*p* < 0.05), indicating that RTE, particularly at medium and high doses, effectively slowed body weight gain in obese mice.

#### 2.3.3. Effects on Lee’s Index and Fat Index

As shown in Table 3, Lee’s index and the fat index were significantly elevated in the model group compared with the normal control group (*p* < 0.05). After treatment with medium and high doses of RTE, both indices decreased significantly relative to the model group (*p* < 0.05), suggesting that RTE improved obesity-related phenotypes.

#### 2.3.4. Effects on the Liver Index

Although the liver index showed an increasing trend in the model group and was partially restored by RTE and Orlistat treatments (Table 4), these differences lacked statistical significance. This static relative ratio likely stems from the simultaneous and proportional decline in both gross body weight and absolute liver weight following RTE administration. Nevertheless, the robust improvements in hepatic lipid deposition observed in HE and Oil Red staining confirm the clear hepatoprotective efficacy of RTE, independent of the macroscopic organ index.

#### 2.3.5. Effects on Lipid Metabolism

**Figure 7:**
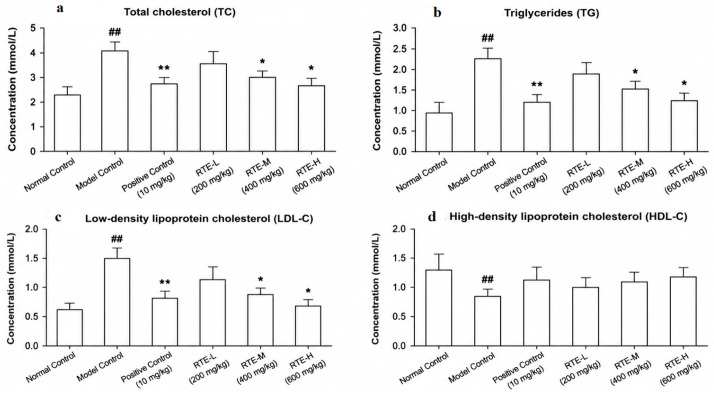
Effects of 60% ethanol-eluted fraction of *R. tanguticum* (RTE) on serum lipid metabolism parameters in high-fat diet (HFD)-induced obese mice. After 8 weeks of intervention, fasting serum levels of (**a**) total cholesterol (TC), (**b**) triglycerides (TG), (**c**) low-density lipoprotein cholesterol (LDL-C), and (**d**) high-density lipoprotein cholesterol (HDL-C) were measured. The treatment groups were administered RTE via oral gavage at low (200 mg/kg), medium (400 mg/kg), and high (600 mg/kg) doses. Data are presented as the Mean ± SD (*n* = 7 per group). Statistical analysis was performed using one-way ANOVA followed by LSD-t or Dunnett’s test. ## *p* < 0.01 compared with the normal control group; * *p* < 0.05 and ** *p* < 0.01 compared with the obesity model control group.

As shown in Figure 7, TC, TG, and LDL-C levels were significantly elevated in the model group, whereas HDL-C was significantly reduced (*p* < 0.05). Medium- and high-dose RTE significantly decreased TC, TG, and LDL-C levels (*p* < 0.05). HDL-C showed an increasing trend in all treatment groups, but the differences were not significant (*p* > 0.05). These results indicate that RTE improved lipid metabolism in obese mice.

#### 2.3.6. Histopathological Evaluation of Liver and Adipose Tissues

**Figure 8:**
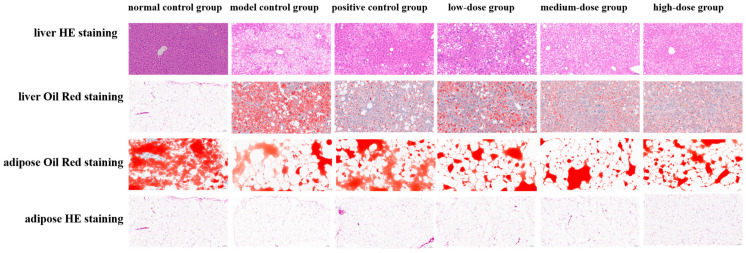
Histopathological evaluation of liver and adipose tissues in high-fat diet (HFD)-induced obese mice treated. Representative morphological images are displayed in rows from top to bottom: hematoxylin and eosin (HE) staining of liver tissues; Oil Red staining of liver tissues; Oil Red staining of adipose tissues; and HE staining of epididymal adipose tissues. The columns represent different experimental groups (from left to right): Normal Control (standard chow diet); Model Control (HFD); Positive Control (HFD + 10 mg/kg orlistat); Low-dose (200 mg/kg); Medium-dose (400 mg/kg); and High-dose (600 mg/kg). Scale bars = 20 μm.

HE staining and Oil Red staining were used to assess histopathological changes in liver tissue and adipocyte morphology. As shown in Figure 8, RTE ameliorated liver injury in a dose-dependent manner. The model group exhibited disorganized hepatic cords, cell swelling, and necrosis, whereas RTE treatment gradually restored tissue integrity and markedly reduced cytoplasmic lipid vacuolization, indicating substantial alleviation of hepatic steatosis and vacuolar degeneration. Oil Red staining further showed that RTE significantly suppressed HFD-induced adipocyte hypertrophy. Compared with the model group, the RTE-treated groups had significantly smaller adipocyte diameters and a corresponding increase in adipocyte number per unit area. Collectively, these findings indicate that RTE effectively improved HFD-induced liver injury and adipose tissue abnormalities in a dose-dependent manner.

## 3. Discussion

Obesity is a complex metabolic disorder characterized not only by an imbalance in energy homeostasis and excessive fat accumulation but also by chronic low-grade inflammation and systemic metabolic dysregulation. While conventional pharmacotherapies (such as orlistat and GLP-1 receptor agonists) achieve significant weight loss, their long-term clinical utility is frequently compromised by adverse effects, including gastrointestinal intolerance and potential cardiovascular risks [21,22]. Consequently, natural botanical extracts with multi-target synergistic properties and favorable safety profiles have emerged as promising alternatives. In the present study, we demonstrated that RTE effectively attenuated body weight gain, ameliorated dyslipidemia, and reversed hepatic steatosis and adipocyte hypertrophy in HFD-induced obese mice, providing robust in vivo evidence for its therapeutic potential. The diverse pharmacological activities of *R. tanguticum* are fundamentally attributed to its complex chemical composition. Our UPLC-QTOF-MS analysis identified 14 major constituents, predominantly anthraquinones and their glycoside derivatives, including emodin, chrysophanol, and rhein. Extensive pharmacological studies have established the metabolic regulatory capacities of these compounds. For example, emodin has been shown to alleviate hepatic steatosis and hyperlipidemia by activating the AMPK signaling pathway and suppressing lipogenic gene expression [23]. Simultaneously, rhein exhibits profound anti-inflammatory properties by inhibiting macrophage infiltration and reducing pro-inflammatory cytokine secretion in adipose tissues [24]. Chrysophanol has also been reported to regulate lipid metabolism and improve insulin resistance [25]. The coexistence of these bioactive anthraquinones in RTE strongly suggests that its anti-obesity efficacy is not reliant on a single molecule, but rather on a synergistic, multi-component interaction that simultaneously targets lipid synthesis, oxidation, and inflammatory cascades. To further decode the systemic molecular mechanisms underlying these phenotypic improvements, we performed an integrated network pharmacology analysis.

The topological analysis identified core hub targets, including TNF, EGFR, and TP53, which are critical mediators of cellular stress, proliferation, and inflammatory responses in metabolic syndrome. More importantly, functional enrichment analysis revealed that the anti-obesity effects of RTE are primarily associated with the PI3K-Akt and MAPK signaling pathways. The PI3K-Akt pathway is a central hub for insulin signaling, glucose uptake, and hepatic lipogenesis; its dysregulation is a hallmark of obesity-induced insulin resistance and fatty liver disease [26]. Meanwhile, the MAPK signaling cascade is intimately involved in adipocyte differentiation, lipid accumulation, and the amplification of chronic inflammatory signals within adipose tissues [27].

Our in vivo histological findings—specifically, the marked reduction in hepatic lipid droplet accumulation and the suppression of adipocyte hypertrophy—physiologically corroborate these bioinformatics predictions, suggesting that RTE may restore metabolic homeostasis by modulating the PI3K-Akt and MAPK signaling networks. Safety is a critical prerequisite for the development of natural products into functional foods or chronic therapeutic agents. In our acute toxicity evaluation, RTE administration at doses up to 5000 mg/kg resulted in no mortality or observable clinical toxicity, classifying the extract as practically non-toxic. This excellent safety profile provides a stark contrast to the frequently reported toxicities of synthetic anti-obesity drugs, highlighting the distinct advantage of *R. tanguticum* for potential long-term metabolic intervention.

Despite these promising findings, the present study has several limitations that warrant critical consideration. First, while network pharmacology provided a robust predictive framework for identifying potential hub targets and pathways, the specific regulatory effects of RTE on the phosphorylation states of key proteins within the PI3K-Akt and MAPK cascades were not directly quantified in vivo. Second, the systemic impact of oral botanical extracts is often mediated through interactions with the gut microbiome, a dimension not explored in the current experimental design. Future investigations utilizing transcriptomics, Western blotting, and specific pathway inhibitors are essential to experimentally validate these molecular targets at the protein level. Additionally, long-term chronic toxicity assessments and 16S rRNA sequencing should be conducted to comprehensively map the safety profile and the gut-liver axis mechanisms of RTE. In summary, this study highlights that RTE exerts significant anti-obesity effects through a synergistic multi-component, multi-target, and multi-pathway network. By ameliorating lipid metabolism disorders and histological abnormalities with high safety margins, *R. tanguticum* presents substantial potential as a functional intervention for obesity and its associated metabolic complications.

In conclusion, the present study demonstrates that the 60% ethanol-eluted fraction of *R. tanguticum* is a safe and effective natural anti-obesity candidate. Through its diverse anthraquinone-rich components, RTE exerts regulatory effects on lipid metabolism and inflammatory responses via multiple targets and signaling pathways, thereby alleviating obesity and associated hepatic injury. These findings not only provide experimental evidence supporting the modern development and utilization of *R. tanguticum* but also establish a scientific foundation for its future application as a functional food, nutraceutical, or adjunctive therapeutic agent for obesity management.

## 4. Materials and Methods

### 4.1. Preparation of RTE

*Rheum* tanguticum Maxim. ex Balf. (10-year-old) was collected from the medicinal plant cultivation base in Donggou Township, Huzhu County, Qinghai Province (altitude 3070 m). The species was authenticated by Researcher Guoying Zhou from the Northwest Institute of Plateau Biology, Chinese Academy of Sciences. A voucher specimen (No. NWIPB-ZGY-06) has been deposited at the Herbarium of Northwest Institute of Plateau Biology, Chinese Academy of Sciences. For extraction, 2 kg of dried *R. tanguticum* was accurately weighed and placed in a tank with 50 L of purified water. After soaking for 1 h, the mixture was refluxed for 2 h. The resulting total extract was concentrated and purified using a ceramic membrane (average pore size 9 nm; Anhui Sanxing Resin Technology Co., Ltd., Bengbu, China) to remove impurities. The purified solution was diluted and passed through a macroporous resin column at a flow rate of 4 BV/h. After adsorption, the column was eluted with 60% ethanol. The eluate was evaporated to dryness in a porcelain dish to obtain the *R. tanguticum* ethanol extract.

### 4.2. Animals

Specific pathogen-free (SPF) male C57BL/6J mice (15–20 g) were purchased from Speifu Biotechnology Co., Ltd. (Wuhan, China) (License No. SCXK 2019-0010). The mice were housed in the animal facility of the Northwest Institute of Plateau Biology in standard individually ventilated cages. Animals were maintained under a strict 12 h light/12 h dark cycle, with a controlled temperature of 25 °C and relative humidity of 50% ± 10%. All mice were acclimatized to the laboratory environment for one week prior to the experiments. High-fat diet and standard maintenance chow were provided by Jiangsu Xietong Pharmaceutical Bio-engineering Co., Ltd. (Nanjing, China). All animal procedures were approved by the Animal Care and Use Ethics Committee of Huaibei University (registration code HBSF-2024-002, approved on 25 March 2024) and conducted in accordance with relevant laboratory animal management regulations.

### 4.3. UPLC-Q-TOF-MS Analysis

#### 4.3.1. Sample Preparation

RTE powder (0.2 g) was accurately weighed into a 50 mL centrifuge tube, and 15 mL of HPLC-grade methanol was added. The mixture was vortexed for 2 min to ensure full dispersion and then subjected to ultrasonic extraction at 40 °C for 30 min (power: 250 W; frequency: 40 kHz). The extract was centrifuged at 9500× *g* (12,000 rpm) for 10 min. The supernatant was collected, filtered through a 0.22 μm microporous membrane, and transferred to an autosampler vial for analysis.

#### 4.3.2. Detection Conditions

Chromatographic separation was performed on a Waters ACQUITY UPLC system equipped with an octadecyl silyl (C_18_) silica column (150 mm × 2.1 mm i.d., 1.8 µm). The mobile phases consisted of 0.1% formic acid in acetonitrile (A) and 0.1% aqueous formic acid (B). The gradient elution profile was: 0–10 min, 5–15% A; 10–30 min, 15–25% A; 30–40 min, 25–40% A; 40–45 min, 40–70% A; 45–50 min, 70–80% A; and 50–60 min, 80–95% A. The detection wavelengths were set at 254, 280, 320, and 360 nm. The flow rate was 0.2 mL/min with an injection volume of 2 µL.

Mass spectrometry was conducted on a Waters Xevo G2-XS QTOF mass spectrometer in both positive and negative ion modes (ESI). The parameters were: scanning range *m*/*z* 100–1500; nebulizer gas: 55 psi; curtain gas: 35 psi; ion source temperature: 600 °C (positive); ion source voltage: 5500 V (positive). For the primary scan, the declustering potential was 100 V, the focusing voltage was 10 V, and the collision-induced dissociation (CID) energies were −20, −40, and −60 V.

### 4.4. Network Pharmacology Prediction

#### 4.4.1. RTE Ingredient Target Selection

The SMILES IDs of compounds in RTE were retrieved from the PubChem database (https://pubchem.ncbi.nlm.nih.gov/), accessed on 15 March 2024. These IDs were imported into SwissTargetPrediction (http://www.swisstargetprediction.ch/), accessed on 15 March 2024, to identify potential protein targets. Targets with a probability score > 0 were selected. All targets were standardized using the UniProt database (https://www.uniprot.org/), and non-human targets were excluded (all accessed in 15 March 2024).

#### 4.4.2. Obesity-Related Target Selection

Human obesity-related genes were identified using the keyword “Obesity” in GeneCards (https://www.genecards.org/), NCBI Gene (https://www.ncbi.nlm.nih.gov), and DisGeNET (https://www.disgenet.org/) databases. After merging the data, R software (version 4.2.1) was used to identify overlapping targets between RTE components and obesity. A Venn diagram was constructed using the “VennDiagram” R package (version 1.7.3) [28].

#### 4.4.3. Protein–Protein Interaction (PPI) Network

The Protein–Protein Interaction (PPI) network of the overlapping targets was constructed using the STRING database and imported into Cytoscape. Topological parameters were calculated using the NetworkAnalyzer plugin. To identify the most critical core hub targets, nodes were ranked based on their topological importance, and the top 10 targets with the highest degree values were selected for further detailed analysis and visualization.

#### 4.4.4. Functional Enrichment and Network Integration

Gene Ontology (GO) enrichment analysis (including biological process (BP), molecular function (MF), and cellular component (CC) and Kyoto Encyclopedia of Genes and Genomes (KEGG) pathway analysis were performed using the STRING database. Terms with an adjusted *p*-value < 0.05 were considered significantly enriched. Bar charts and bubble plots were generated using the R “clusterProfiler” package (version 4.6.2). Finally, a “component–target–disease–pathway” network was constructed using Cytoscape (version 3.8.0).

### 4.5. Animal Experiments

#### 4.5.1. Grouping and Model Induction

Male mice were randomly assigned to six groups (n = 7 per group): normal control (NC), HFD-induced model (Model), positive drug control (Orlistat, 10 mg/kg F. Hoffmann-La Roche Ltd., Basel, Switzerland), and low-, medium-, and high-dose RTE groups (200, 400, and 600 mg/kg, respectively). The NC group was fed standard chow, while all other groups received an HFD (60% kcal from fat; Shulaibao Biotechnology Co., Ltd., Wuhan, China) to induce obesity. The entire experimental period lasted for 8 weeks. Simultaneously with HFD induction, the treatment groups received RTE suspension via oral gavage. Throughout the study, daily monitoring procedures were implemented to record food intake, water consumption, and general behavioral changes of the mice.

#### 4.5.2. Acute Toxicity Test

Twenty SPF mice (10 males, 10 females) were acclimated for one week. They were randomly divided into groups and administered RTE suspension via oral gavage at doses of 1000, 2000, 3000, 4000, and 5000 mg/kg. In accordance with the OECD Guidelines for the Testing of Chemicals (Acute Oral Toxicity), all animals were strictly monitored for mortality, behavioral changes, and any overt signs of clinical toxicity at least once daily throughout a 14-day observation period. The median lethal dose 50%(LD50) and its 95% confidence interval were calculated using the Karber method.

#### 4.5.3. Organ Indices and Physical Parameters

Food intake was recorded daily, and body weight was measured every 3 days. After the final treatment, mice were fasted overnight. Body weight (g) and body length (nose-to-anus, cm) were measured. The Lee’s index, liver index, and adipose-to-body weight ratio were calculated as follows:

Lee’s index = (^3^√Body weight (g) × 10)/Length (cm)

Liver index (%) = (Liver weight/Body weight) × 100%

Adipose ratio (%) = Perigonadal fat weight/Body weight

#### 4.5.4. Biochemical Analysis

In week 9, after a 12 h fast, mice were anesthetized via ether inhalation. Blood (approx. 1.2 mL) was collected from the retro-orbital plexus and centrifuged at 10,000 rpm for 15 min to separate serum. Levels of triglycerides (TG), total cholesterol (TC), low-density lipoprotein cholesterol (LDL-C), and high-density lipoprotein cholesterol (HDL-C) were determined using commercial kits (Shanghai Keaibo Biotechnology Co., Ltd., Shanghai, China).

#### 4.5.5. Histopathological Analysis

Liver and epididymal adipose tissues were harvested. Liver tissues were fixed in 4% paraformaldehyde and subsequently embedded in paraffin, and sectioned at a thickness of 5 μm for hematoxylin and eosin (HE) staining. Adipose tissues were embedded in OCT compound, snap-frozen, and then cryosectioned for Oil Red staining.

For H&E staining, paraffin sections were deparaffinized in xylene, rehydrated through a graded ethanol series, and stained with hematoxylin for 3 min. After differentiation in hydrochloric acid–ethanol and bluing under running tap water, sections were dehydrated through a graded ethanol series and stained with eosin for 5 min. Following further dehydration, sections were cleared in xylene, mounted with neutral balsam, and examined under a light microscope [29,30].

For Oil Red staining, frozen sections were air-dried briefly at room temperature, fixed (e.g., in 4% paraformaldehyde for 10 min), and rinsed with distilled water. Sections were then stained with Oil Red solution in the dark for 8–10 min, differentiated in 60% ethanol until the background became colorless, and the reaction was stopped by rinsing in distilled water. Nuclei were lightly counterstained with hematoxylin, blued under running tap water, washed with water, and mounted with glycerol gelatin for microscopic observation [31]. Stained sections were examined and photographed under a light microscope (Olympus BX53, Evident Corporation, Tokyo, Japan]) at 200× magnifications. Digital images were captured using cellSens Standard software (Version 4.5).

### 4.6. Statistical Analysis

Data were analyzed using SPSS v19.0 and expressed as Mean ± Standard Deviation (SD). For comparisons between two groups, the independent samples *t*-test was used. For multiple groups, one-way ANOVA followed by LSD-t or Dunnett’s test was used for data with normal distribution and homogeneous variance [32]. For non-normal data, non-parametric tests were applied. If variances were unequal, Tamhane’s T2 or Dunnett’s T3 tests were used. *p* ≤ 0.05 was considered statistically significant.

## 5. Conclusions

In conclusion, the 60% ethanol-eluted fraction of *R. tanguticum* exhibits significant, safe anti-obesity effects in HFD-fed mice by reducing weight gain, improving dyslipidemia, and attenuating hepatic and adipose tissue steatosis. UPLC-QTOF-MS and network pharmacology identified anthraquinone derivatives as the primary active constituents, which likely mediate these benefits via the MAPK and PI3K-Akt signaling pathways. However, a limitation of this study is the reliance on computational target predictions without direct experimental validation. Therefore, future studies utilizing Western blotting, transcriptomics, or specific pathway inhibitors are required to explicitly confirm these molecular mechanisms. Overall, RTE is a promising multi-target natural candidate for obesity management.

## Figures and Tables

**Table 1:** Mass spectrometry information of 60% ethanol-eluted fraction of *R. tanguticum* (RTE).

Time (min)	Molecular (*m*/*z*)	Fragments	Compound
16.47	431.0992	293, 269	Emodin-1-O-glucoside
19.81	461.1097	169, 147	Galloyl-1-O-cinnamoyl-6-O-glucoside
20.52	431.0986	269, 241	Emodin-3-O-glucoside
25.34	407.1350	245	Torachrysone-8-O-glucoside
25.76	415.1039	253	Chrysophanol-1-O-glucoside
27.07	415.1039	253	Chrysophanol-8-O-glucoside
27.37	431.0985	269, 241	Emodin-8-O-glucoside
28.04	431.0984	323, 161	Emodin-6-O-glucoside
33.19	445.1147	283	Physcion-8-O-glucoside
34.23	477.1038	313	Isolindleyin
35.19	477.1038	313, 169	Lindleyin
38.95	283.0251	239	Rhein
40.00	607.1826	443	Physcion-8-O-diglucoside
40.97	607.1833	443	Physcion-1-O-diglucoside

**Table 2:** Body weight changes of mice in each group.

Group	Initial Weight (Week 1)	Final Weight (Week 9)
Normal Control	26.27 ± 1.74	29.46 ± 2.35
Obesity Model control	27.02 ± 3.07	56.76 ± 2.64 ^#^
Positive Control	26.64 ± 2.64	34.13 ± 2.53 **
60%-L	26.40 ± 3.16	49.12 ± 1.77
60%-M	26.14 ± 2.63	42.34 ± 1.26 *
60%-H	26.06 ± 3.15	38.88 ± 0.56 *

Note: Compared with normal control group, ^#^ *p* < 0.01; Compared with obesity model control group, * *p* < 0.05, ** *p* < 0.01.

**Table 3:** Lee’s index and fat-to-body ratio of each group of mice.

Group	Lee’s Index	Fat-to-Body Ratio
Normal Control	2.83 ± 1.24	1.26 ± 0.43
Obesity Model Control	3.96 ± 1.38 ^#^	1.98 ± 0.37 ^#^
Positive Control	3.17 ± 1.05 *	1.45 ± 0.58
60%-L	3.79 ± 1.19	1.86 ± 0.52
60%-M	3.40 ± 1.22	1.61 ± 0.39
60%-H	3.25 ± 1.24 *	1.51 ± 0.39 *

Note: Compared with normal control group, ^#^ *p* < 0.05; Compared with obesity model control group, * *p* < 0.05.

**Table 4:** The liver coefficients of each group of mice.

Group	Liver Coefficients (%)
Normal Control	2.67 ± 1.04
Obesity Model Control	3.54 ± 1.12
Positive Control	2.98 ± 1.57
60%-L	3.39 ± 1.64
60%-M	3.32 ± 1.39
60%-H	3.28 ± 1.76

## Data Availability

The original contributions presented in this study are included in the article. Further inquiries can be directed to the corresponding authors.
